# LINC00483 Has a Potential Tumor-Suppressor Role in Colorectal Cancer Through Multiple Molecular Axes

**DOI:** 10.3389/fonc.2020.614455

**Published:** 2021-01-20

**Authors:** Duilia Brex, Cristina Barbagallo, Federica Mirabella, Angela Caponnetto, Rosalia Battaglia, Davide Barbagallo, Rosario Caltabiano, Giuseppe Broggi, Lorenzo Memeo, Cinzia Di Pietro, Michele Purrello, Marco Ragusa

**Affiliations:** ^1^ Department of Biomedical and Biotechnological Sciences – Section of Biology and Genetics “Giovanni Sichel,” University of Catania, Catania, Italy; ^2^ Department of Medical, Surgical Sciences and Advanced Technologies G.F. Ingrassia, University of Catania, Catania, Italy; ^3^ Department of Experimental Oncology, Mediterranean Institute of Oncology (IOM), Catania, Italy

**Keywords:** long non-coding RNAs, microRNAs, colorectal cancer, competing endogenous RNA, epithelial–mesenchymal transition, HNF4α, TGFβ-1, IL-6

## Abstract

Long non-coding RNAs (lncRNAs) are the most heterogeneous class of non-protein-coding RNAs involved in a broad spectrum of molecular mechanisms controlling genome function, including the generation of complex networks of RNA-RNA competitive interactions. Accordingly, their dysregulation contributes to the onset of many tumors, including colorectal cancer (CRC). Through a combination of *in silico* approaches (statistical screening of expression datasets) and *in vitro* analyses (enforced expression, artificial inhibition, or activation of pathways), we identified LINC00483 as a potential tumor suppressor lncRNA in CRC. LINC00483 was downregulated in CRC biopsies and metastases and its decreased levels were associated with severe clinical features. Inhibition of the MAPK pathway and cell cycle arrest by starvation induced an upregulation of LINC00483, while the epithelial to mesenchymal transition activation by TGFβ-1 and IL-6 caused its down-modulation. Moreover, enforced expression of LINC00483 provoked a slowing down of cell migration rate without affecting cell proliferation. Since LINC00483 was predominantly cytoplasmic, we hypothesized a “miRNA sponge” role for it. Accordingly, we computationally reconstructed the LINC00483/miRNA/mRNA axes and evaluated the expression of mRNAs in different experimental conditions inducing LINC00483 alteration. By this approach, we identified a set of mRNAs sharing the miRNA response elements with LINC00483 and modulated in accordance with it. Moreover, we found that LINC00483 is potentially under negative control of transcription factor HNF4α. In conclusion, we propose that LINC00483 is a tumor suppressor in CRC that, through an RNA-RNA network, may control cell migration and participate in proliferation signaling.

## Introduction

Colorectal cancer (CRC) is the most common malignancy in the digestive system and the fourth leading cause of cancer-related death around the world, mainly from tumor metastasis (1). Although modern oncology has made great efforts to shed light on the underlying molecular mechanisms of the development of CRC, much of it is still unclear. Accordingly, it is needed to explore new regulatory mechanisms of CRC onset and progression in order to improve diagnostics and the development of innovative therapeutic strategies. The Vogelstein’s model, based on a sequence of molecular changes within protein-coding genes leading to cancer transformation, is now considered too simplistic to explain the complex heterogeneity of cancer, including and especially CRC (2, 3). Notably, only less than 2% of the human genome encodes proteins, while about 85% is pervasively transcribed in RNAs other than mRNAs, denominated non-coding RNAs (ncRNAs), including microRNAs (miRNAs) and long non-coding RNAs (lncRNAs) (4–6). LncRNAs are more than 200 nt in length with no or limited protein-coding power and account for a large part of the human genome (7). Although lncRNAs were initially considered as unfunctional transcriptional “noise,” recent studies have revealed that they play a key regulatory role in multiple cellular processes, including stem cell pluripotency, apoptosis, cell differentiation, migration, genomic stability, and epithelial–mesenchymal transition (EMT) (8). Accordingly, it is not surprising that lncRNA dysregulation plays a very important role in tumorigenesis indeed, the oncogenic or tumor-suppressive action of lncRNAs has been largely demonstrated to participate in cancer transformation and progression (9, 10). Through binding DNA, RNAs, and proteins, lncRNAs exhibit a broad spectrum of molecular mechanisms by which they influence gene expression, such as chromatin modification, RNA transcription, pre-mRNA splicing, mRNA translation, and protein localization (11, 12). In the last few years, several pieces of evidence have been collected on the involvement of lncRNAs in CRC onset and progression. LncRNAs affect critical CRC signaling pathways by acting both as oncogenes and tumor suppressors through interactions with other regulatory molecules (13–15). Several lncRNAs have been reported to be dysregulated in CRC, suggesting promising potentiality for theranostic applications (16–18). However, the molecular mechanisms of action in CRC pathobiology were elucidated only for few of them, such as, CCAT1, CCAT2, H19, HOTAIR, MALAT1, and UCA1 (19–25). One of the most explored molecular mechanisms of lncRNAs in cytoplasm is their activity as miRNA sponges. The ability of lncRNAs to sequester miRNAs by sequence complementarity has important systemic effects on the RNA network inside cells. Indeed, the hypothesis of competing endogenous RNA (ceRNA) foresees that lncRNAs and mRNAs, sharing the same miRNA response elements (MREs), compete for binding to same miRNAs, regulating each other’s expression (26, 27). According to the hypothesis that the expressions of lncRNAs and mRNAs with the same MREs would be positively and negatively correlated to each other and to miRNAs, respectively, in this paper we propose a combined approach of *in silico* and experimental biology to identify an lncRNA whose dysregulated expression is associated with CRC pathobiology. More specifically, based on the hypothesis that RNA-RNA network functioning is grounded on relative stoichiometric concentrations of interacting RNA molecules, our aim was the identification and characterization of a lncRNA whose expression was linearly related to that of mRNAs dysregulated in association with most serious clinical features of CRC patients. Following its computational identification, LINC00483 was experimentally analysed to understand its involvement in CRC and its associated molecular axes.

## Material and Methods

### Computational Analysis

We retrieved from “R2 Genomics” (https://hgserver1.amc.nl/cgi-bin/r2/main.cgi?&species=hs) datasets containing expression data on CRC (**Table S1**) in order to identify differentially expressed (DE) genes associated with the most severe features of the tumor phenotype (e.g., metastases, microsatellite instability, advanced tumor, node, metastasis [TNM] stage, KRAS, BRAF and TP53 mutations, CpG island methylator phenotype [CIMP] status). Results were filtered by p-value (≤ 0.01), using the ANOVA statistical test and the “false discovery rate” as correction criterion for multiple tests. From each dataset we recovered genes that were downregulated/upregulated in CRC, according to the above-mentioned clinical-pathological features.

By overlapping gene lists (downregulated/upregulated gene lists) retrieved from each dataset, we obtained a single list of genes showing the same type of dysregulation (i.e., up- or down-regulation) in at least 50% of consulted datasets. We employed these CRC deregulated genes as BGs to identify CRC related lncRNAs. More specifically, we selected those lncRNAs showing positive or negative correlation with BGs by calculating the Pearson coefficient (p-value < 0.01) between the expression values of the BGs and the expression values of all lncRNAs in each dataset previously analyzed. We compared the lists of lncRNAs associated with each BG for each dataset and selected only those lncRNAs shared by at least 50% of datasets.

**Figure 1:**
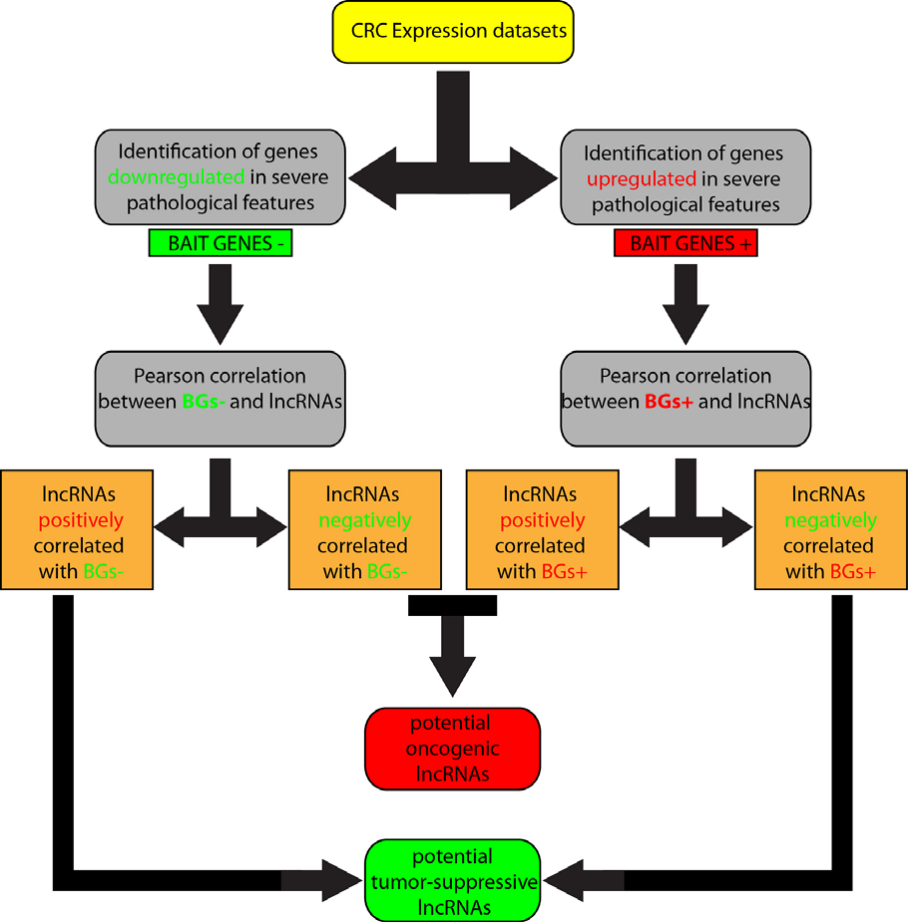
Workflow of the computational approach followed to identify l*ong non-coding RNAs* (lncRNAs) associated to *colorectal cancer* (CRC).

Finally, we generated 4 lists of lncRNAs correlated with BGs associated with the most severe clinical-pathological features of CRC:

1. lncRNAs showing positive correlation with downregulated genes;
2. lncRNAs showing positive correlation with upregulated genes;
3. lncRNAs showing negative correlation with downregulated genes;
4. lncRNAs showing negative correlation with upregulated genes;

We overlapped lists 1–4, and lists 2–3, because we speculated that lncRNAs positively correlated with downregulated genes corresponded roughly with the list of lncRNAs negatively correlated with upregulated genes and, in the same way, lncRNAs positively correlated with upregulated genes were in the list of lncRNAs negatively correlated with downregulated genes. Therefore, lncRNAs shared by the two compared lists were selected. By this approach, we obtained i) a list of potential tumor-suppressor lncRNAs by overlapping of 1 and 4, and ii) a list of potential oncogene lncRNAs by overlapping of 2 and 3 (**Figure 1**).

### Long Non-Coding RNA Target Identification

Since one of the most common functions of lncRNAs is that of “miRNA sponge,” we identified miRNAs that could simultaneously bind the selected lncRNAs and the genes positively correlated with them. In this supposed “miRNA sponge” model, the lncRNA and mRNA should exhibit a mutual positive expression correlation, while the miRNA—acting as a “trait d’union” negative regulator RNA—should have a negative expression correlation with the same lncRNA and mRNA (**Figure S1**). To investigate this hypothesis, we retrieved miRNAs showing a negative correlation with the studied lncRNAs. MiRNA expression values were retrieved from TCGA colon cancer (COAD) (n=261), through UCSC XENA (https://xenabrowser.net/datapages/), and compared to lncRNA expression from the same dataset. We calculated the Pearson coefficient between miRNA and lncRNA expression values and selected miRNAs showing a negative correlation with the selected lncRNAs (p-value ≤0.001). We simultaneously retrieved miRNAs harboring binding sites for lncRNAs through LncBase (http://carolina.imis.athena-innovation.gr/diana_tools/web/index.php?r=lncbasev2%2Findex-experimental), miRcode (http://www.mircode.org/), and RNA22 v.2 tool (https://cm.jefferson.edu/rna22/Interactive/) and completed these outputs with those resulting from correlation analysis. To complete the molecular axis of the lncRNA-miRNA-mRNA interaction, we identified mRNAs targeted by these miRNAs. We interrogated the datasets selected in the early step of the study in order to identify those mRNAs that showed the strongest positive correlation with lncRNAs, as previously described. For these mRNAs were verified: a) the binding to miRNAs previously identified through TarBase v.8 (http://carolina.imis.athena-innovation.gr/diana_tools/web/index.php?r=tarbasev8%2Findex); b) their negative expression correlation with the same miRNAs (TCGA COAD). To make this analysis more effective and focused on our pathological model, it was performed just on those miRNAs showing an involvement in CRC by using mirCancer (http://mircancer.ecu.edu/) and mir2Disease (http://www.mir2disease.org/) databases.

### Cell Lines

HCT-116 cell lines, derived from primary colon tumors, were obtained from the Interlab Cell Line Collection (ICLC), an “International Repository Authority” within the IRCCS Azienda Ospedaliera Universitaria San Martino-IST Istituto Nazionale per la Ricerca sul Cancro (Genova, Italia). HCT-116 cells were maintained in Roswell Park Memorial Institute-1640 (RPMI-1640) medium (Gibco), supplemented with 10% fetal bovine serum (FBS) (Gibco), 1% 2 mM L-glutammine (Lonza, Basel, Switzerland), and 1% penicillin/streptomycin (10,000 U/ml) (Gibco). Cells were cultured at 37°C and 5% CO2.

### RNA Isolation From Cell Lines

Total RNA was extracted from cell lines with TRIzol^®^ (Invitrogen, Carlsbad, CA, USA), according to the manufacturer’s instructions, and quantified by GenQuant Pro Spectrophotometer (Biochrom) and Qubit fluorescence quantification system (Invitrogen). Isolated RNA was treated with DNase, according to the manufacturer’s instructions. The RNA Subcellular Isolation Kit (Active Motif) was used to analyze lncRNA expression separately in the nucleus and cytoplasm of HCT-116 cells. The lncRNAs MALAT1 and TUG1 were used as markers of nuclear and cytoplasmic fractions, respectively.

### RNA Isolation From Colorectal Cancer Formalin-Fixed, Paraffin-Embedded Samples

Twenty CRC patients were recruited from “Azienda Ospedaliero—Universitaria Policlinico Vittorio Emanuele” (Catania, Italy). Formalin-Fixed, Paraffin-Embedded (FFPE) tumor tissues and adjacent normal colon mucosa were isolated at the Section of Anatomic Pathology, Department G.F. Ingrassia, University of Catania (Catania, Italy) (**Table 1**). RNA was extracted from FFPE samples through the PureLink FFPE RNA Isolation Kit (Invitrogen), according to the manufacturer’s instructions, and quantified by GenQuant pro spectrophotometer (Biochrom) and Qubit (Invitrogen).

**Table 1:** Colorectal cancer (CRC) biopsies analyzed in this study.

Cohorts	Mean age (years ± Std Dev)	Sex		Grading		pT		pN		M	
		M	F	G1/G2	G3	T1/T2	T3/T4	N= 0	N > 0	M0	M1
OCT samples	68 ± 12	19	16	31	4	7	28	20	15	31	4
FFPE samples	69 ± 11	11	9	15	5	5	15	13	7	20	0

### RNA Isolation From Colorectal Cancer Tissues Embedded in OCT and Fresh Biopsies

A second independent group of 35 CRC samples embedded in Optimal Cutting Temperature (OCT) were analyzed. All samples were provided from “Istituto Oncologico del Mediterraneo (IOM)” Viagrande (Catania, Italy) (**Table 1**). OCT tumor tissues were sliced with a cryostat at the Section of Anatomic Pathology, Department G.F. Ingrassia, University of Catania (Catania, Italy). Before RNA extraction, samples were treated to remove OCT as follows:

1. Wash OCT tissue samples three times with 500 µl DEPC H2O; centrifuge 4°C x 5 min at 11,000 g (Eppendorf Centrifuge 5424 R) and remove supernatant after each step;
2. Wash with 500 µl ETOH 25%; centrifuge 4°C x 5 min at 11,000 g (Eppendorf Centrifuge 5424 R) and remove supernatant.

Total RNA was extracted from OCT tissue samples and from fresh biopsies through the PureLink FFPE RNA Isolation Kit (Invitrogen) and TRIzol ^®^ (Invitrogen, Carlsbad, CA, USA), respectively, according to the manufacturer’s instructions. Isolated RNA was quantified by GenQuant pro spectrophotometer (Biochrom).

### PCR Primer Design

We designed specific PCR primers for the selected lncRNAs and their target mRNAs by using the online tool Primer-Blast (http://www.ncbi.nlm.nih.gov/tools/primer-blast/). Specific PRC primers were also designed for the housekeeping gene PPIA (peptidylprolyl isomerase A), used for normalization. Primer pairs are shown in **Table S2**.

### Expression Analysis by Real-Time PCR

We investigated the expression of the selected lncRNAs and mRNA axis in CRC cell lines and CRC patient tissues through Real Time PCR, by using Power SYBR Green RNA-to-CT 1-Step Kit (Applied Biosystems, Thermo Fisher Scientific, Waltham, MA, USA), according to the manufacturer’s instructions. All reactions were performed on a 7900HT Fast Real-Time PCR System (Applied Biosystems). We used PPIA as reference gene, both for cell lines and CRC tissues. DE gene fold changes were calculated by applying the 2-ΔΔCt method. A paired T-test was used to compare FFPE sample ΔCts, while an unpaired T-test was used for evaluate all the other analyses. P-value ≤0.05 was established as statistically significance.

### HCT-116 Transfection With LINC00483

We transfected the HCT-116 cell line with pcDNA3.1(+) expression vector to induce LINC00483 overexpression. The cDNA sequence of LINC00483 was cloned into pcDNA3.1(+) by EcoRV, consistent with the direction of the CMV promote. The empty pcDNA3.1(+) vector was used as a scramble molecule. Both constructs were synthesized and purchased from the GenScript company. Ten micrograms of plasmids were re-suspended in 40 µl TE buffer 1X. Cells (2 x 105 per well) were seeded in 24-well plates and transfected with 750 ng plasmid together with LipofectamineTM 2000 (Invitrogen) by using the direct transfection method, according to the manufacturer’s instructions. Cells were harvested at 48 h after transfection and lysed with TRIzol for RNA extraction. Total RNA was used both to confirm transfection efficiency (>90%) and to evaluate the expression of axis’s mRNAs by Real Time PCR. All experiments were performed in biological triplicates.

### Functional Assays

The CCK-8 assay was performed to assess cellular viability/proliferation at 48 h AT, according to the manufacturer’s instructions. Absorbance values were read with Varioskan™ LUX (Thermo Scientific™). We transfected HCT-116 cell lines with an expression vector and its respective scramble molecule by seeding 2.5 x 104 cells per well in 96-well plates and appropriately scaling the amount of transfection reagents. All experiments were performed in biological triplicates.

Migration rates after transfection were evaluated by OrisTM Universal Cell Migration Assembly kit (Platypus Technologies), according to the manufacturer’s instructions. Briefly, we transfected HCT-116 cells with the expression vector and its scramble molecule by seeding 3.5 x 104 cells per well in 96-well plates—after insertion of Oris Cell Seeding Stoppers—and appropriately scaling the amount of transfection reagents. To create the detection zone at the center of the well, the stoppers were removed at 24 h AT (0 h). Detection zones for each well were photographed with a microscope, Leitz FLUOVERT (Leica Microsystems), at 0 h (premigration reference wells) and at 24, 48, 72, 96, and 144 h AT. Cells that migrated into the detection zone were quantified through the ImageJ software package. This analysis allowed us to quantify cells that had migrated into the detection zone for all the time points. All experiments were performed in biological triplicates.

### TGFβ-1 Treatment

We performed an *in vitro* EMT by treatment with TGFβ-1, a cytokine secreted by tumor cells and stromal fibroblasts in the tumor microenvironment and considered a primary inducer of EMT (28). 5 x 104 cells per well were seeded in 24-well plates and maintained in serum starvation conditions (0.5% FBS) for 24 h. Successively, cells were treated with 20 ng/ml TGFβ-1 for 24 h. Control samples were maintained in RPMI-1640 medium (Gibco) supplemented with an equal volume of solvent. All experiments were performed in biological triplicates.

### Interleukin-6 Treatment

We treated HCT-116 cell line with IL-6, a multifunctional cytokine whose signaling hyper-activation is associated with tumor onset and development. 3 x 104 cells per well were seeded in 24-well plates and maintained in serum starvation conditions (0.5% FBS) for 24 h. Successively, cells were treated with 200 ng/ml IL-6 and exposed for 24 and 48 h. Control samples were maintained in RPMI-1640 medium (Gibco) supplemented with an equal volume of solvent. All experiments were performed in biological triplicates.

### Treatment With MAPK Inhibitor U0126

We performed a pharmacological inhibition of the MAPK pathway by treating the HCT-116 cell line with U0126, a highly selective ATP-non-competitive MEK1/2 inhibitor, which specifically prevents *in vitro* phosphorylation of MEK1/2 by binding to the inactive enzyme and blocking ERK recruitment. HCT-116 cells were seeded (3.2 x 104 per well) in 24-well plates and cultured in serum starvation conditions (no FBS) for 24 h; successively, cells were treated with 25 µM U0126 (MEK1/2 inhibitor, Merck, Darmstadt, Germany) and exposed to the drug for 12 and 24 h. Control samples were treated with an equivalent volume of DMSO (solvent of the drug used for treatment). All experiments were performed in biological triplicates.

### Cell Cycle Arrest

To investigate if the expression of the selected lncRNAs is modulated during artificial cell cycle arrest, we evaluated HCT-116 response to serum starvation. 5 x 105 cells were seeded in 24-well plates. We separately treated two groups of samples: the first group of samples was exposed to serum starvation by maintaining cells in RPMI-1640 medium (Gibco) (no FBS) for 24 h. Control samples were maintained in RPMI-1640 (10% FBS). A second group of cells was synchronized by serum starvation (no FBS) for 24 h and subsequently incubated with fresh medium with 10% FBS for a further 24 h. Control samples were incubated with fresh RPMI-1640 without serum. All experiments were performed in biological triplicates.

### Treatment With HNF4α Inhibitor BI6015

To verify the transcriptional regulation of LINC00483 by HNF4α, we treated the HCT-116 cell line with BI6015, a specific inhibitor of this transcription factor. 8 x 104 cell were seeded in 24-well plates and treated with 80 µM BI6015 (Focus Biomolecules) for 24 h. Control samples were treated with an equivalent volume of DMSO (solvent). All experiments were performed in biological triplicates.

### Identification of Transcription Factor Binding Sites

Potential transcription factor binding sites (TFBSs) harbored on LINC00483 promoter and upstream regulatory region (1 kb) were retrieved from ENCODE tracks (i.e., transcription factor ChIP-seq clusters, DNaseI hypersensitivity clusters) and UCSC regulatory tracks (i.e., regulatory elements from ORegAnno, SwitchGear genomics transcription start sites) mapped on UCSC Genome Browser (https://genome.ucsc.edu/).

## Results

### Screening of Colorectal Cancer Expression Datasets, Long Non-Coding RNA Selection

By following the approach reported in methods, we identified from each CRC expression dataset those genes that were downregulated/upregulated in CRC, according to the specified clinical-pathological features.

By overlapping gene lists (downregulated/upregulated gene lists) retrieved from each dataset, we obtained a single list of upregulated genes (CYP1B1, NPR3, RGL2, SLIT2, TSPAN2) and a single list of downregulated genes (ACOT7, AGPAT5, ATP5B, AURKAIP1, CASP1, CEP55, CXCL3, FUT4, GSR, HNRNPAB, IDO1, KIF11, MCM5, PBK, PIGR, RANBP1, SCO2, TOE1, TTLL12) showing the same trend of expression alteration in at least 50% of datasets.

These CRC deregulated genes were used as “bait genes” (BGs) to identify lncRNAs showing a statistically significant positive correlation (PC) or negative correlation (NC) of expression with BGs.

We compared the lists of lncRNAs associated with each BG from each dataset and selected only those lncRNAs shared by at least 50% of datasets.

Finally, we generated four lists of lncRNAs (**Table 2**) correlated with BGs:

1. lncRNAs showing PC with downregulated genes (**Table 2**, column A);
2. lncRNAs showing PC with upregulated genes (**Table 2**, column B);
3. lncRNAs showing NC with downregulated genes (**Table 2**, column C);
4. lncRNAs showing NC with upregulated genes (**Table 2**, column D);

**Table 2:** Long non-coding RNAs (LncRNAs) positively/negatively correlated with BGs.

Positive correlation (PC)		Negative correlation (NC)	
A	B	C	D
DLEU1	LINC00312	A2M-AS1	DLEU1
EP300-AS1	MEG3	ADAMTS9-AS2	LINC00261
HCP5	RUNX1-IT1	ASAP1-IT1	LINC00483
LINC00261	TP73-AS1	CCDC144NL-AS1	TMPO-AS1
LINC00483		CCDC18-AS1	LINC01207
LINC00675		DAPK1-IT1	MCF2L-AS1
LINC01207		DLEU2	
MCF2L-AS1		EGOT	
TFAP2A-AS1		HYMAI	
THAP9-AS1		KLF3-AS1	
TTC28-AS1		LINC00312	
USP30-AS1		LINC00622	
		LINC00623	
		LINC00663	
		LINC00702	
		LINC00865	
		LINC00869	
		LINC00893	
		LINC01138	
		LINC01279	
		LINC01410	
		LINC-PINT	
		MAGI2-AS3	
		MEG3	
		MIR99AHG	
		MLLT4-AS1	
		NR2F1-AS1	
		PSMA3-AS1	
		PSMD5-AS1	
		RUNX1-IT1	
		SERTAD4-AS1	
		STX17-AS1	
		TAPT1-AS1	
		TP53TG1	
		TP73-AS1	
		TUG1	
		ZNF561-AS1	
		ZNF667-AS1	

LncRNAs showing PC with downregulated BGs are shown in column A. LncRNAs showing PC with upregulated BGs are shown in column B. LncRNAs showing NC with downregulated BGs are shown in column C. LncRNAs showing NC with upregulated BGs are shown in column D.

Finally, as reported in methods, we overlapped these lists, in order to obtain a set of potential tumor-suppressor lncRNAs (i.e., DLEU1, LINC00261, LINC00483, LINC01207, MCF2L-AS1) and oncogene lncRNAs (MEG3, RUNX1-IT1, and TP73-AS1) to experimentally analyze.

**Figure 2:**
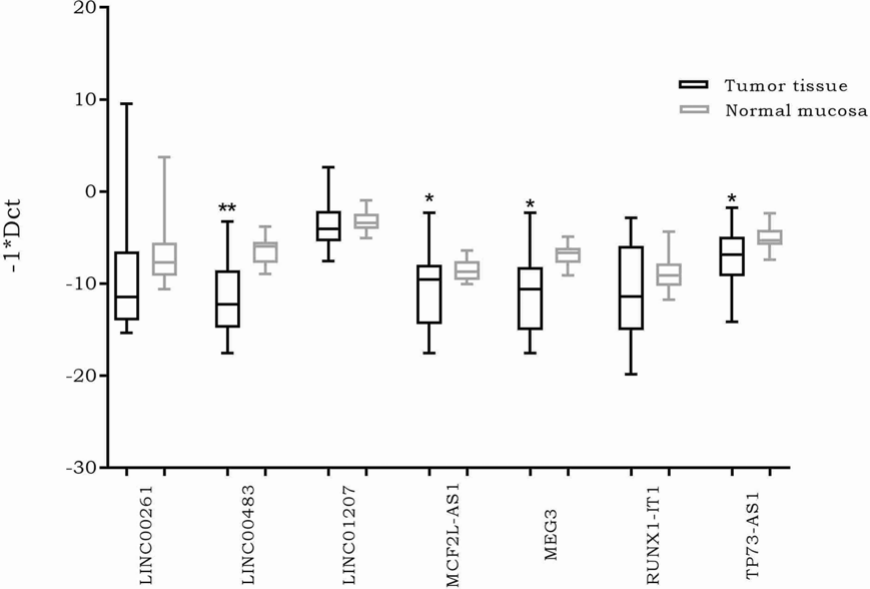
Box plots showing differential expression of the selected long non-coding RNAs (lncRNAs) in 20 *formalin-fixed, paraffin-embedded* (FFPE) colorectal cancer *(*CRC) biopsies compared to normal adjacent tissues. Different primer pairs for DLEU1 did not produce any amplification. For this reason, DLEU1 data are not shown. *: p-value < 0.05; **: p-value < 0.001.

The expression of the previously selected lncRNAs was investigated through real time PCR in 20 formalin-fixed, paraffin-embedded (FFPE) CRC biopsies and their normal adjacent tissues (NATs). The results showed a significantly differential expression of four lncRNAs. More specifically, MEG3, MCF2L-AS1, LINC00483, and TP73-AS1 were downregulated in CRC tumor tissues compared to NATs (**Figure 2**).

### Long Non-Coding RNA Expression After TGFβ-Induced Epithelial–Mesenchymal Transition in HCT-116 Cells

**Figure 3:**
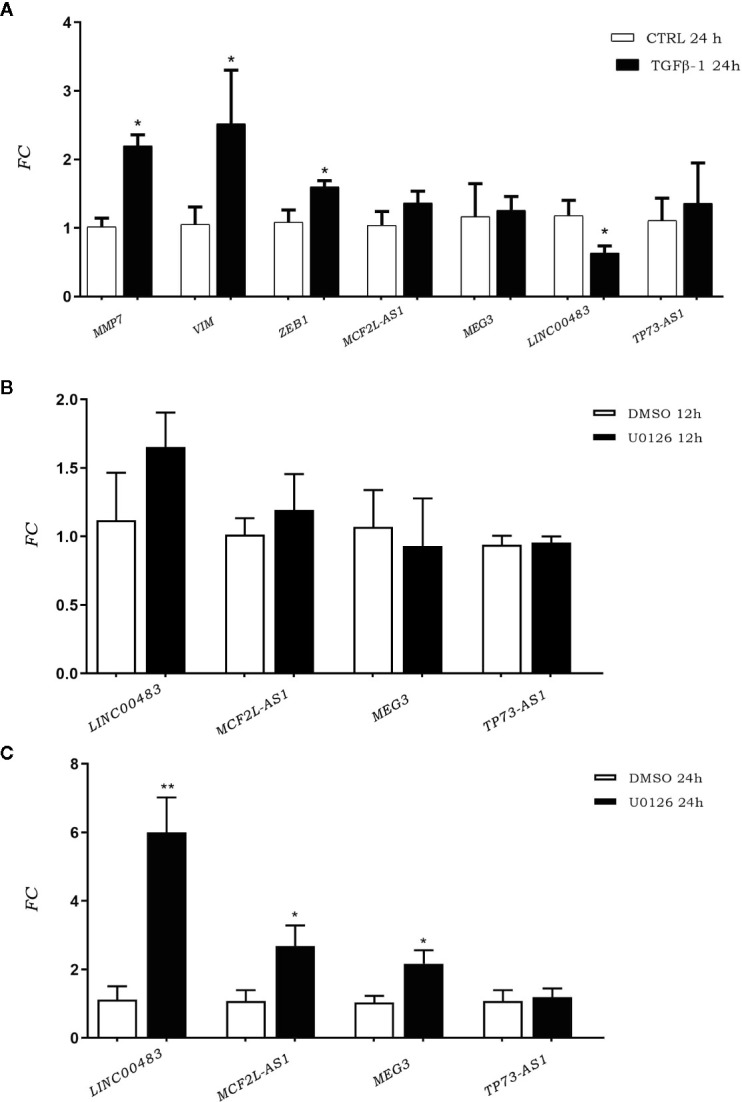
**(A)** Expression of epithelial–mesenchymal transition (EMT) gene markers and DE long non-coding RNA (lncRNA) expression after TGFβ-1 treatment. **(B)** LncRNA expression in HCT-116 cell line post treatment with U0126 12 h and 24 h **(C)**. *p-value<0.05; **p-value<0.01.

To investigate the involvement of previously identified differentially expressed lncRNAs (DE lncRNAs) in cellular proliferation and metastases, we induced the EMT through TGFβ-1 (transforming growth factor beta 1). We investigated TGFβ-1 effects on HCT-116 cells analyzing the expression of DE lncRNAs 24 h after TGFβ-1 treatment. We assessed the expression of EMT gene markers (MMP7, VIM, and ZEB1) to verify the successful outcome of *in vitro* TGFβ-1 treatment. Results showed an increased expression of MMP7, VIM, and ZEB1 at 24 h after treatment (AT) (**Figure 3A**), in agreement with mesenchymal transformation. Moreover, the results showed that TGFβ-1 treatment significantly decreased the expression of lncRNA LINC00483 at 24 h AT, whereas the expression of two other lncRNAs (MCF2L-AS1, and TP73-AS1) increased, even if in a statistically non-significant way (**Figure 3A**).

### Inhibition of MAPKs Affects Long Non-Coding RNA Expression

We performed an *in vitro* inhibition of the MAPK pathway in order to investigate the involvement of previously identified DE lncRNAs in proliferative signaling. LncRNA expression was assessed in HCT-116 cells at 12 and 24 h AT with the MAPK inhibitor U0126. Results showed that the expression of DE lncRNAs was affected by the inhibition of the MAPK pathway (**Figures 3B, C**). More specifically, U0126 treatment did not alter the lncRNA expression after 12 h of treatment (**Figure 3B**), however, MCF2L-AS1, MEG3, and LINC00483 significantly increased their expression at 24 h AT, although the strongest upregulation was observed for LINC00483 (**Figure 3C**).

### LINC00483 Affects Cell Migration but Not Cell Proliferation

**Figure 4:**
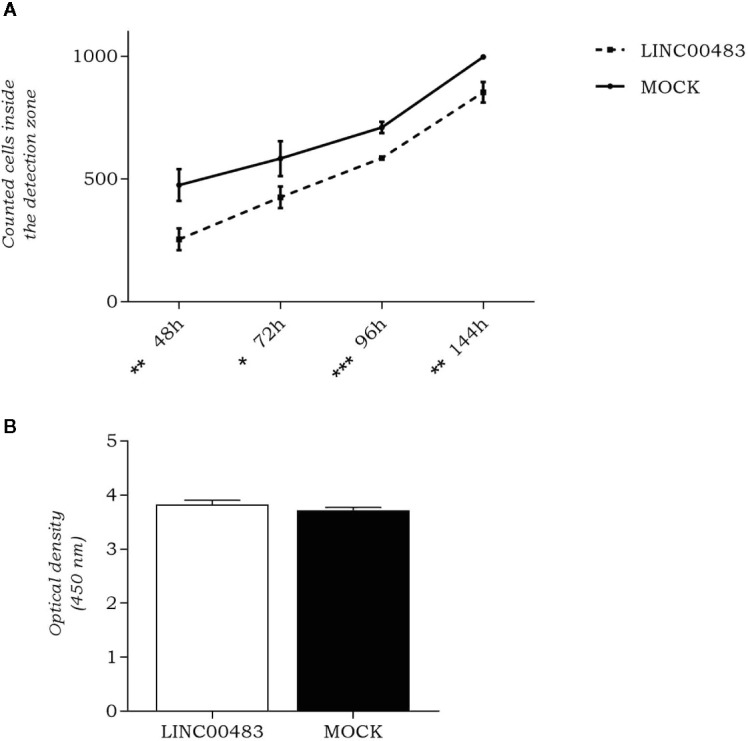
LINC00483 functional assays. **(A)** Number of cells migrated into the detection zone in transfected HCT-116 cells compared to negative controls (MOCK), at 48, 72, 96, and 144 h after transfection. *p-value < 0.05; **p-value < 0.01; ***p-value < 0.001; **(B)** CCK-8 assay was performed to assess HCT-116 cell proliferation at 48 h after LINC00483 transfection.

As LINC00483 expression was altered by *in vitro* modulation of proliferation and EMT, we evaluated whether its enforced expression affected cellular migration and cell count. By OrisTM Universal Cell Migration Assembly kit (Platypus Technologies), we counted the cells migrated into the detection zone at different time points: 48, 72, 96, and 144 h after cell seeding stopper removal. Results showed a significant decrease of migration rate in LINC00483-transfected HCT-116 cells compared to negative controls (MOCK), at all specified time points, but the most statistically significant difference was observed at 96 h after transfection (**Figure 4A**). On the other hand, the cell viability/proliferation assay 48 h after LINC00483 transfection did not show any significant difference between transfected HCT-116 cells and their negative controls (**Figure 4B**). Taken together, the data on functional assays suggest that the upregulation of LINC00483 directly impaired cell migration but not cell viability/proliferation.

### LINC00483 Molecular Axis Identification

Our previous results revealed a significant downregulation of LINC00483 in FFPE CRC biopsies compared to adjacent normal mucosa; furthermore, LINC00483 expression decreased in HCT-116 cells treated with TGFβ-1, while it increased when MAPK signaling was inhibited. Accordingly, we focused our next experimental analyses on LINC00483 due to its consistent data in biopsies and *in vitro* treatments. We screened all the datasets (1,313 datasets) deposited on R2 Genomics for LINC00483 dysregulation (**Table 3**) and found an almost specific downregulation of LINC00483 in CRC; moreover, its decreased expression was frequently associated with bad prognosis. Taken together all these data suggest a potential tumor suppressor role for LINC00483.

**Table 3:** LINC00483 dysregulation in several expression datasets.

Dataset	Class	Expression	*P-value*
GSE3629 (121)	Class: colorectal cancer *vs*. non-neoplastic rectal mucosa	Down in tumor	1.50E−19
GSE3629 (121)	Group: sporadic ca *vs*. uc-nonca	Down in sporadic ca	1.50E−17
GSE21510 (148)	Tissue: cancer-lcm *vs*. normal-homogenized	Down in cancer	5.80E−16
GSE8671 (64)	Tissue: adenoma *vs*. normal	Down in adenoma	4.70E−10
GSE20916 (145)	Tissue: adenocarcinoma *vs*. normal_colon	Down in adenocarcinoma	1.20E−09
GSE3629 (121)	Group: sporadic ca *vs*. uc-ca	Down in sporadic ca	1.50E−09
GSE76124 (198)	tumor_grade: poorly_differentiated *vs*. well_differentiated	Down in poorly differentiated	5.70E−08
GSE20916 (145)	Malignancy: carcinoma vs distant_normal_colon	Down in carcinoma	4.50E−07
GSE8671 (64)	nt_size: 0.2_cm_(x3) *vs*. 2.0_cm	Down in 2 cm	4.60E−07
GSE35896 (62)	braf_mutation: n *vs*. y	Down in y	5.30E−07
GSE8671 (64)	nt_size: 0.2_cm_(x3) *vs*. 1.5_cm	Down 1.5 cm	8.60E−07
GSE2109 (315)	nt_pathological_grade: 2 *vs*. 3	Down in 3	3.10E−06
GSE39582 (566)	cimp_status: neg *vs*. pos	Down in pos	3.30E−06
GSE35896 (62)	braf_mutation: n *vs*. y	Down in y	7.50E−06
GSE20916 (145)	Tissue: carcinoma *vs*. normal_colon	Down in carcinoma	1.90E−05
GSE6791 (84)	sample_source: cervical normal *vs*.c head and neck cancer	Down in cancer	1.90E−05
GSE6791 (84)	Type: cervical normal *vs*. head and neck cancer	Down in cancer	1.90E−05
GSE4554 (84)	ms_status: msi *vs*. mss	Down in msi	2.00E−05
GSE8671 (64)	nt_location_tissue: sigmoid_colon_adenoma *vs*. sigmoid_colon_normal	Down in adenoma	2.60E−05
GSE76124 (198)	tumor_grade: moderately_differentiated *vs*. well_differentiated	Down in moderately differentiated	3.80E−05
GSE23878 (59)	Group: colon tumor *vs*. normal paired tissue	Down in colon tumor	4.70E−05
GSE29271 (210)	survival_time_(months): 35 *vs*. 9	Down in 35	5.60E−05
GSE20916 (145)	Malignancy: adenoma *vs*. carcinoma	Down in adenoma	6.10E−05
GSE13294 (155)	microsatellite_status: msi *vs*. mss	Down in msi	1.20E−04
GSE8671 (64)	nt_location_tissue: descending_colon_adenoma *vs*. rectum_normal	Down in adenoma	5.30E−04
GSE8671 (64)	nt_location_tissue: descending_colon_adenoma *vs*. descending_colon_normal	Down in adenoma	5.40E−04
GSE20916 (145)	Tissue: colon_tumor *vs*. norma_colon	Down in tumor	6.10E−04
GSE3629 (121)	Group: uc-associated ca *vs*. uc-nonca	Down in ca	6.90E−04
GSE41258 (390)	Tissue: normal colon *vs*. polyp	Down in polyp	2.20E−03
GSE17538 (232)	ajcc_stage: 2 *vs*. 4	Down in 2	4.20E−03
GSE35896 (62)	pten_mutation: n *vs*. y	Down in y	4.20E−03
GSE29271 (210)	survival_time_(months): 36 *vs*. 6	Down in 36	4.60E−03
GSE42363 (14)	tumor_grade: moderately *vs*. poorly	Down in poorly diffentiated	5.20E−03
GSE4183 (53)	Group: colon_adenoma *vs*. healthy_control	Down in adenoma	5.20E−03
GSE35896 (62)	pten_mutation: n *vs*. y	Down in y	5.40E−03
GSE50948 (156)	invasive_tumor_area_size: 15 *vs*. 6	Down in 15	5.80E−03
GSE33114 (108)	sample_descr: normal colon mucosa vs primary tumor resection	Down in tumor	8.30E−03
GSE33114 (108)	sample_source: normal colon mucosa *vs*. primary tumor resection	Down in tumor	8.30E−03
GSE33114 (108)	Type: normal *vs*. tumor	Down in tumor	8.30E−03
GSE21510 (148)	Tissue: cancer-homogenized *vs*. normal-homogenized	Down in cancer	8.30E−03
GSE4107 (22)	Tissue: mucosa_control *vs*. mucosa_patient	Down in patients	8.60E−03
GSE9891 (285)	Stagecode: ia *vs*. iii	Down in ia	9.50E−03
GSE35896 (62)	kras_mutation: n *vs*. y	Down in no	9.60E−03
GSE17538 (232)	ajcc_stage: 1 *vs*. 2	Down in 2	9.70E−03

In this table are reported 1) GEO ID (the sample number is shown in brackets); 2) class of comparison; 3) LINC00483 expression; 4) p-values of ANOVA test for each dataset.

**Figure 5:**
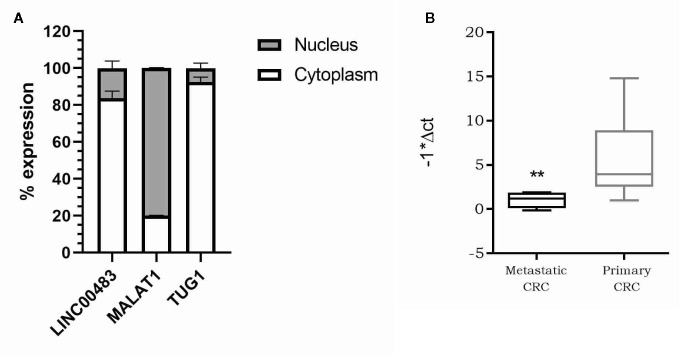
**(A)** Nucleus/cytoplasm distribution of LINC00483. LncRNAs MALAT1 and TUG1 are analyzed as markers of nuclear and cytoplasmic localization, respectively; **(B)** Box plot showing LINC00483 expression in metastatic colorectal cancers (CRCs) compared to non-metastatic primary tumors. **p-value < 0.01.

As the molecular functions of lncRNAs are strictly linked to their localization inside the cell, we first investigated the subcellular localization of LINC00483. We isolated RNA from the nuclear and cytoplasmic fractions of HCT-116 cells and analyzed LINC00483 in both, together with MALAT1, predominantly nuclear, and TUG1, preferentially located in the cytoplasm. Our data showed that LINC00483 is predominantly cytoplasmic (**Figure 5A**). Accordingly, we hypothesized a “miRNA sponge” role for LINC00483 and retrieved miRNAs that could simultaneously bind LINC00483 and the mRNAs showing a positive correlation of expression with LINC00483. More specifically, we selected miRNAs showing a negative correlation of expression with LINC00483 by using the TCGA COAD dataset (**Table S3**). We also retrieved miRNAs harboring binding sites for LINC00483 through LncBase, miRcode, and the RNA22 v.2 tool and overlapped these outputs with those resulting from correlation analysis (**Table S3**).

For each previously selected miRNA of **Table S3**, we identified the experimentally validated mRNA targets through TarBase v.8. This analysis did not produce any results for the following miRNAs: miR-4464, miR-4660, miR-4743-3p, and miR-7978, which were excluded from the next analyses. We also verified that the couples miRNA:mRNA target from TarBase showed a negative expression correlation (TCGA COAD). Moreover, to make this analysis more effective and focused on our cancer model, including a tumor-suppressor lncRNA, we considered just those miRNAs showing a potential involvement in CRC as oncomiRNAs by using mirCancer and mir2Disease databases. Finally, we filtered the list of miRNA targets with a set of mRNAs showing a positive correlation of expression with LINC00483 in several CRC datasets, as described in “*Material and Methods*.” By this approach, we obtained a list of potential LINC00483:miRNA:mRNA molecular axes. **Table 4** shows miRNAs harboring binding sites for LINC00483, which also show a negative expression correlation with LINC00483. For each miRNA, mRNA targets (positively correlated with LINC00483) are shown. The table has been filtered for mRNAs showing the highest R-values (ACVR1B, ADD3, ARL4A, CC2D1A, CD9, CTNNA1, DSP, EIF6, EPCAM, GALNT3, HIGD2A, MIER3, PLS1, PRKAA1, RNF43, SH3YL1, SRPK1, TPD52, USP7, VDR, and ZDHHC9). Data including all miRNA targets and their correlations with LINC00483 are reported in **Tables S4**-**S11**.

**Table 4:** Predicted LINC00483 molecular axes in *colorectal cancer* (CRC).

miRNA	miRNA expression (mirCancer/mir2Disease)	mRNA target	miRNA/mRNA correlation (panCancer)	LINC00483/mRNA correlation
miR-30a-5p	Downregulated	DSP	−0.11 (2.00E−02)	0.406 (2.68E−12)
		PLS1	−0.185 (7.57E−05)	0.569 (7.92E−26)
		TPD52	−0.271 (5.39E−09)	0.428 (1.02E−13)
miR-205-3p	Downregulated	PRKAA1	−0.098 (3.81E−02)	0.207 (2.27E−04)
miR-302c-3p	Downregulated	ARL4A	−0.087 (6.47E−02)	0.471 (7.75E−17)
		VDR	−0.072 (1.25E−01)	0.559 (1.61E−30)
miR-330-5p	Downregulated	ACVR1B	−0.106 (2.47E−02)	0.442 (1.14E−04)
		CC2D1A	−0.206 (1.09E−05)	0.32 (1.02E−07)
		HIGD2A	−0.158 (7.55E−04)	0.363 (5.07E−12)
		RNF43	−0.238 (3.37E−07)	0.451 (2.56E−15)
		ZDHHC9	−0.102 (2.98E−02)	0.389 (3.10E−11)
miR511-5p	Downregulated	SH3YL1	−0.084 (6.27E−01)	0.349 (3.84E−119
		USP7	−0.122 (9.56E−03)	0.372 (2.78E−10)
miR-544a	Upregulated	CD9	0.014 (7.73E−01)	0.391 (1.89E−10)
		DSP	−0.007 (8.80E−01)	0.406 (2.68E−12)
		EPCAM	−0.019 (6.95E−01)	0.479 (1.92E−17)
		GALNT3	0.041 (3.84E−01)	0.422 (2.66E−13)
		MIER3	0.015 (7.46E−01)	0.376 (1.53E−10)
miR-1231		CTNNA1	−0.238 (3.21E−07)	0.321 (2.00E−09)
		EIF6	−0.045 (3.45E−01)	0.44 (1.68E−14)
miR-3619-5p		ACVR1B	−0.133 (4.64E−03)	0.442 (1.14E−04)
		ADD3	−0.02 (6.76E−01)	0.437 (1.10E−17)
		SRPK1	−0.063 (1.81E−01)	0.38 (1.00E−10)

Expression in CRC, mRNA targets, mRNAs/miRNA correlations, mRNAs/LINC00483 correlations are reported for each miRNA negatively correlated with LINC00483 and harboring binding sites for it.

### LINC00483 Expression in Colorectal Cancer Tissues Embedded in OCT and Fresh Biopsies

Our previous analysis on FFPE CRC biopsies showed a statically significant downregulation of LINC00483 in tumor tissues compared to normal mucosa. To corroborate these data and understand the potential LINC00483 association with clinical features of CRC patients, we assessed its expression on a second independent cohort of 35 CRC samples embedded in OCT (optimal cutting temperature). LINC00483 was significantly downregulated in metastatic CRCs compared to non-metastatic primary tumors (**Figure 5B**). We found no dysregulation of LINC00483 in association with other specific sub-groups of samples based on tumor and node staging and tumor grade.

### Effects of Forced Expression of LINC00483 on the mRNA Axis

**Figure 6:**
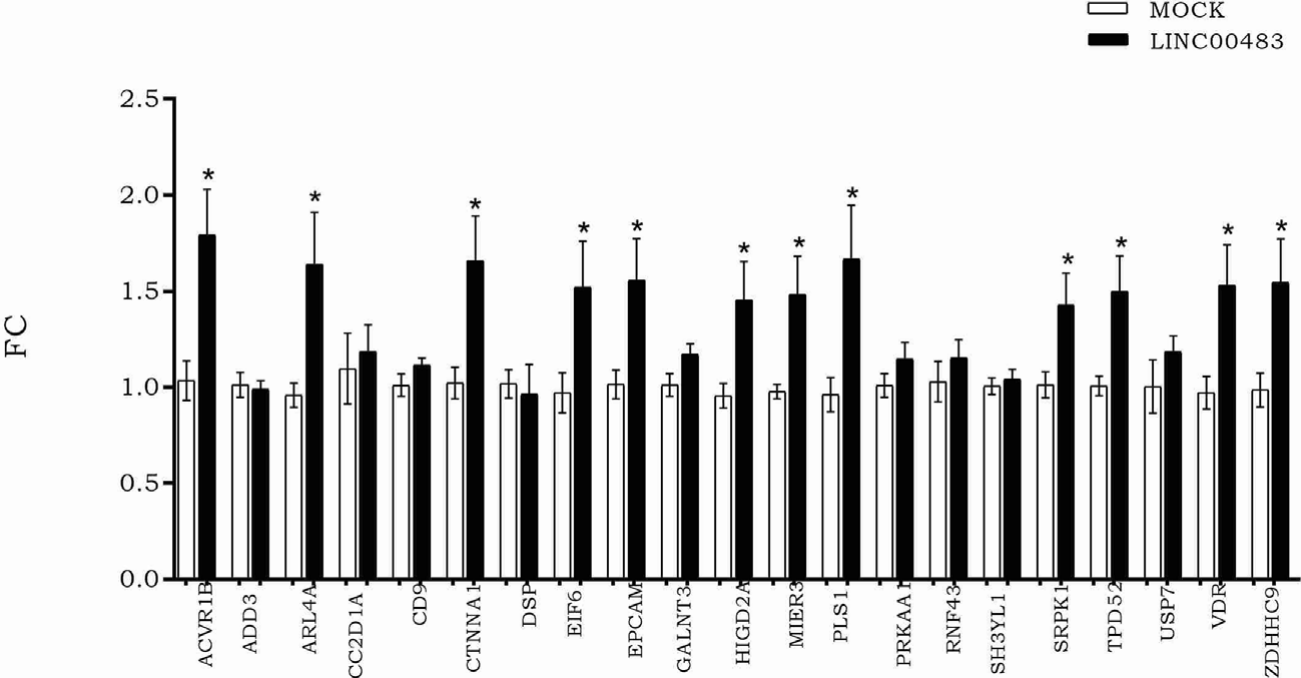
The expression of messenger RNAs (mRNAs) selected as potentially involved in LINC00483 molecular axes after LINC00483 transfection in HCT-116 cells. *p-value < 0.05.

According to LINC00483 low expression in tumors tissues, we induced its *in vitro* overexpression by transfecting HCT-116 cells with the expression vector pcDNA3.1, including the LINC00483 cDNA sequence, in order to verify how the expression of mRNA nodes of the axes was modulated (**Figure 6**). The expression of 12 mRNAs statistically increased in HCT-116 cells transfected with LINC00483 compared to control samples, transfected with the scramble molecule (MOCK): ACVR1B, ARL4A, CTNNA1, EIF6, EPCAM, HIGD2A, MIER3, PLS1, SRPK1, TPD52, VDR, and ZDHHC9.

### Inhibition of MAPKs Affected mRNA Expression of LINC00483 Axis

**Figure 7:**
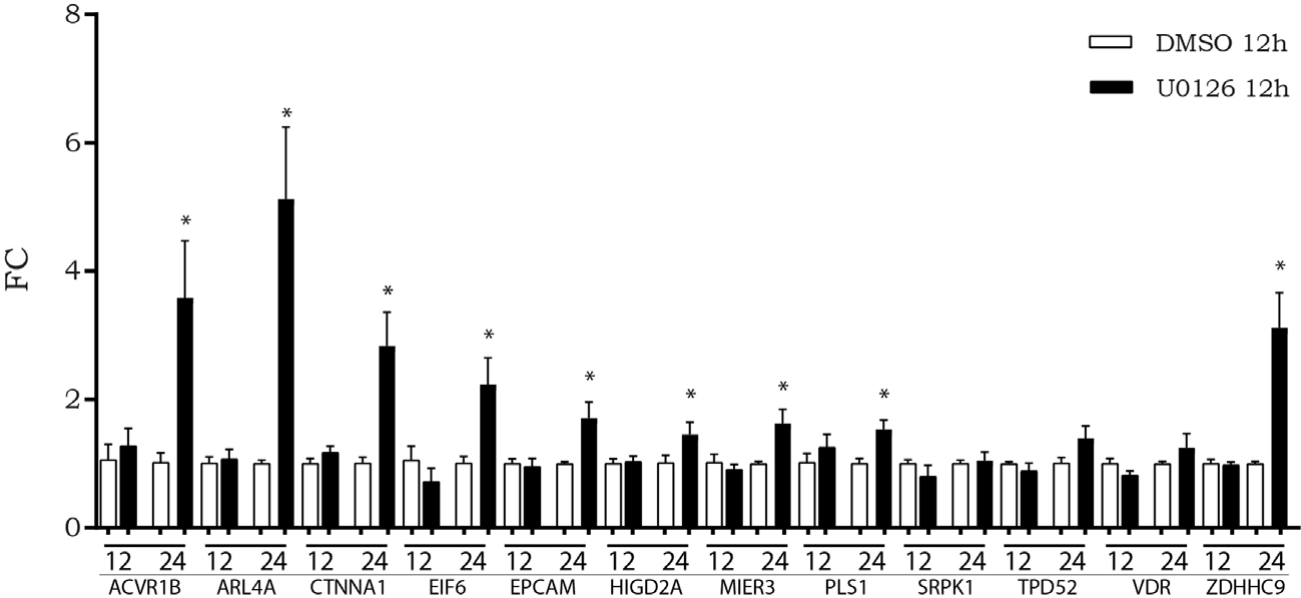
Expression of messenger RNAs (mRNAs) of LINC00483 molecular axes in HCT-116 cells at 12 and 24 h after U0126 treatment. *p-value < 0.05.

We previously observed that, after *in vitro* inhibition of the MAPK pathway, LINC00483 expression increased at 12 h AT, although a statistically significant variation was observed at 24 h AT. Accordingly, we evaluated if the expression of mRNAs showing increasing expression after LINC00483 enforced expression was altered by U0126 treatment. The expression of nine mRNAs statistically increased at 24 h AT: ACVR1B, ARL4A, CTNNA1, EIF6, EPCAM, HIGD2A, MIER3, PLS1, ZDHHC9 (**Figure 7**).The results would show that most of LIN00483-regulated mRNAs after MAPK blockage exhibited an expression modulation following that of LINC00483, thus suggesting the existence of a potential molecular relationship among these RNAs.

### Expression of LINC00483 and the mRNA Axis After Cell Cycle Arrest

**Figure 8:**
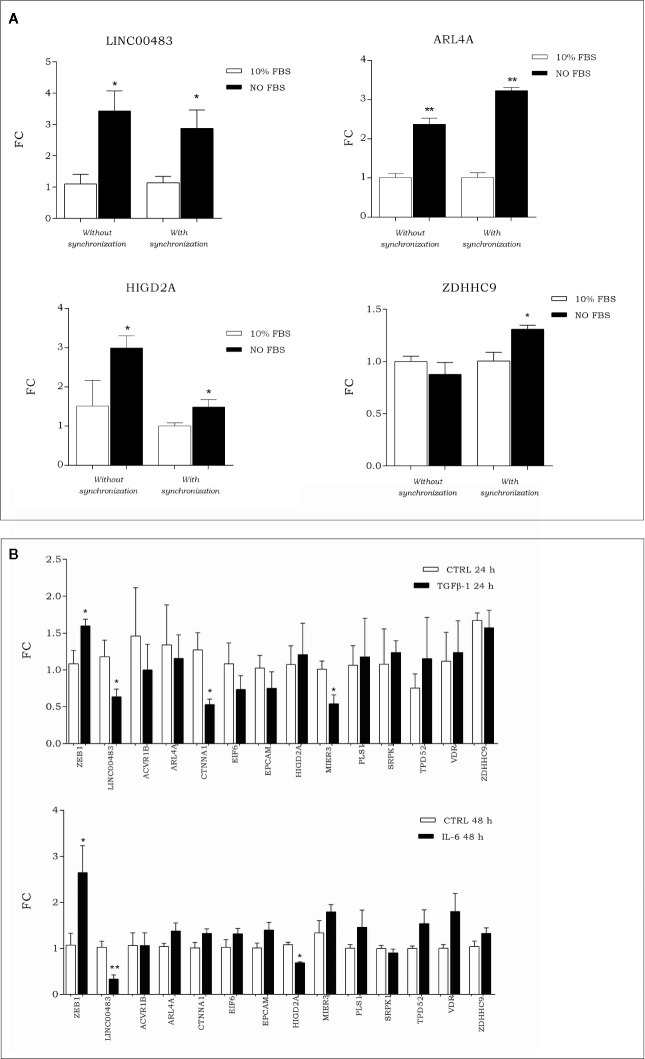
**(A)** Expression of LINC00483 and messenger RNAs (mRNAs) associated with its molecular axes after 24 h serum starvation, with and without cell synchronization. To make this panel more readable, we reported just significant data. The complete panel of histograms is reported in **Figure S2**. **(B)** Expression of EMT marker ZEB1, LINC00483 and mRNAs potentially involved in the LINC00483 axes after TGFβ-1 and Il-6. *p-value ≤ 0.05; **p-value ≤ 0.01.

To investigate how the expression of LINC00483 and its target genes was modulated during artificial cell cycle arrest without chemical inhibitors, we exposed HCT-116 cells to serum starvation. More specifically, we assessed the expression of LINC00483 and mRNAs (ACVR1B, ARL4A, CTNNA1, EIF6, EPCAM, HIGD2A, MIER3, PLS1, SRPK1, TPD52, VDR, and ZDHHC9) in two different ways: 1) starved cells (0% FBS) *vs*. 10% FBS cells; 2) starved cells (0% FBS) *vs*. 10% FBS cells after cell synchronization obtained by 24 h of serum starvation. The expression of LINC00483 significantly increased after 24 h serum starvation, with and without cell synchronization. Interestingly, two mRNAs belonging to the LINC00483 axes (ARL4A and HIGD2A) showed statistically significant expression variations similar to LINC00483, while ZDHHC9 was upregulated only in starved cells compared to 10% FBS cells after cell synchronization (**Figure 8A**). These data would suggest again that, similar to MAPK inhibition, growth arrest induced by serum starvation activated the expression of LINC00483. However, in this specific cell condition only the expression of three mRNAs was induced, suggesting that the activation of LINC00483 axes depends on functioning of specific cellular pathways.

### Expression of LINC00483 and Axes’s mRNAs After In Vitro Epithelial–Mesenchymal Transition

To assess the involvement of LINC00483 and mRNAs potentially linked to it in metastasis-related mechanisms, we induced *in vitro* the EMT by treating HCT-116 cells with TGFβ-1 and IL-6 (interleukin-6). Both these treatments induced a downregulation of LINC00483 (**Figure 8B**). TGFβ-1 treatment showed a statistically significant downregulation for CTNNA1 and MIER3 while IL-6 provoked the downregulation of HIGD2A (**Figure 8B**). Notably, CTNNA1, HIGD2A, and MIER3 showed the same expression trends as LINC00483 in the same model of *in vitro* EMT. These data would show that two different signaling of EMT induction similarly repressed LINC00483. As previously observed, also in this case different cellular signaling could control different LINC00483 molecular axes.

### HNF4α Transcription Factor Potentially Controls LINC00483 Expression

**Figure 9:**
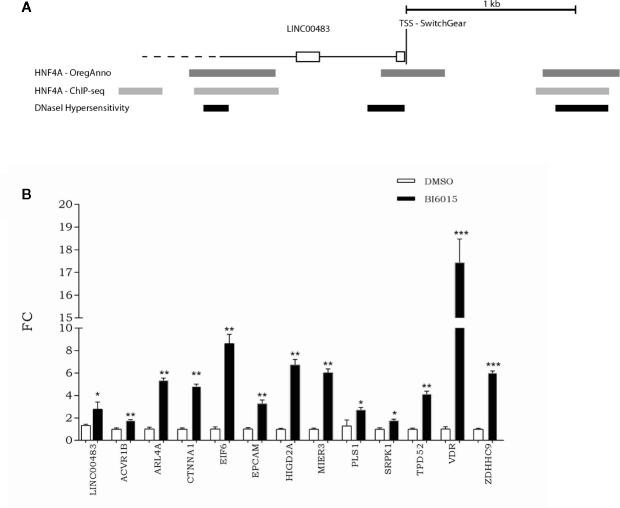
Relationship between LINC00483 and the HNF4α transcription factor. **(A)** HNF4α binding sites and DNase hypersensitive regions upstream and downstream of the transcription start site (TSS) of LINC00483; **(B)** Expression of LINC00483 and messenger RNAs (mRNAs) potentially involved in LINC00483 axis at 24 h after treatment with BI6015, an HNF4α inhibitor. *p-value ≤ 0.05; **p-value ≤ 0.01; ***p-value ≤ 0.001.

To get a better insight into transcriptional regulation of LINC00483, we screened the UCSC Genome Browser to find potential transcription factor binding sites (TFBSs). More specifically, by overlapping different tracks derived from ENCODE experiments to the UCSC Genome browser, we found TFBSs for HNF4α (hepatocyte nuclear factor 4α) about 180 nt upstream of the transcription start site (TSS) in correspondence with a DNase hypersensitive region (**Figure 9A**). Moreover, other HNF4α TFBSs were present about 1 kb upstream and downstream of TSS overlapping DNase hypersensitive regions (**Figure 9A**). These data suggest that HNF4α could contribute to transcriptional regulation of LINC00483. Indeed, we treated CRC cells with BI6015, an HNF4α inhibitor, and evaluated the expression of LINC00483 and the axis’s mRNAs. Chemical inhibition of HNF4α induced the significant upregulation of LINC00483 at 24 h after BI6015 treatment (**Figure 9B**), suggesting a potential negative control by HNF4α on LINC00483 transcription. Notably, the expression of all mRNAs strongly increased at 24 h after treatment (**Figure 9B**), corroborating our previous data concerning the positive expression association between LINC00483 and the mRNAs belonging to the LINC00483:miRNA:mRNA axes.

### CTNNA1, HIGD2A, MIER3

**Figure 10:**
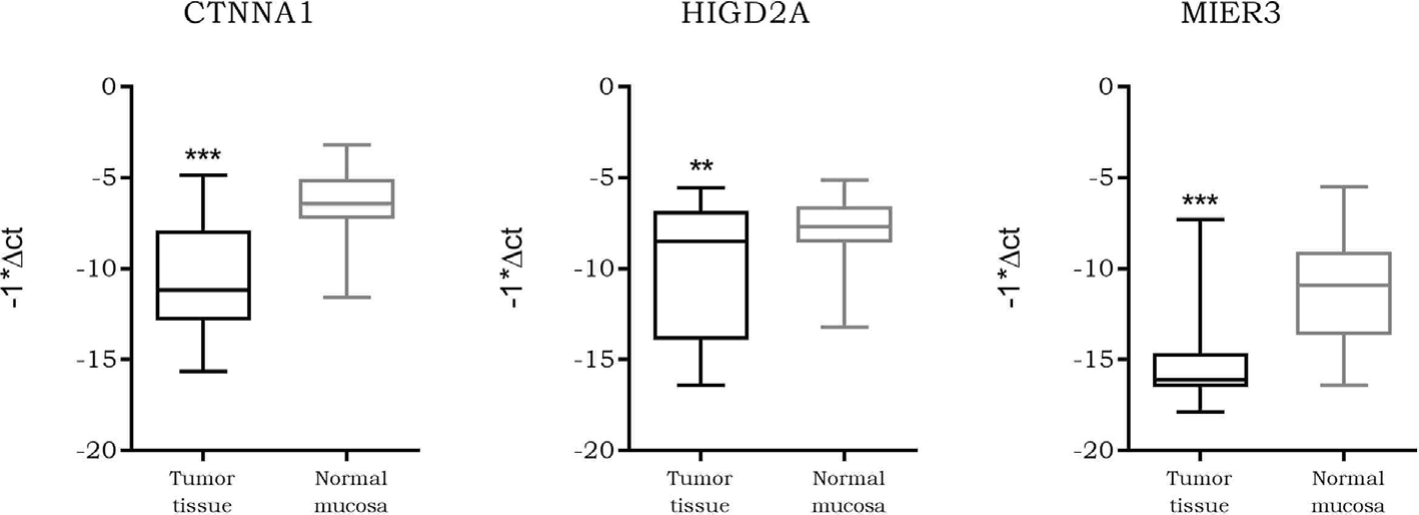
Box plot showing the differential expression of CTNNA1, HIGD2A, and MIER3 in *colorectal cancer* (CRC) formalin-fixed, paraffin-embedded *(*FFPE) biopsies compared to normal adjacent mucosa. **p-value ≤0.01, ***p-value ≤0.001.

As our data suggested a tighter association of expression between LINC00483 and CTNNA1, HIGD2A, and MIER3 with respect the other axis’s mRNAs, we further analyzed them. We screened the expression of CTNNA1, HIGD2A, and MIER3 in all CRC datasets deposited on R2 genomics. The results showed their stable downregulation in CRC tumor tissues compared to controls, or, in most cases, in association with the severe features of the tumor (**Table S12**). We investigated CTNNA1, HIGD2A, and MIER3 expression in CRC biopsies compared to adjacent normal mucosa in order to confirm their downregulation in CRC observed by dataset screening: the three mRNAs showed a statistically significant downregulation in tumor tissues compared to normal mucosa (**Figure 10**). Moreover, we calculated the Pearson coefficients between the expression values of LINC00483 and CTNNA1, HIGD2A, and MIER3 in the same CRC samples, obtaining significant positive correlation of expression between LINC00483 and CTNNA1 (r-value: 0.47, p-value: 0.03), LINC00483 and HIGD2A (r-value: 0.41, p-value: 0.05), LINC00483 and MIER3 (r-value: 0.53, p-value: 0.01). Positive correlations between LINC00483 and CTNNA1, HIGD2A, and MIER3 was corroborated by the analysis of expression correlation performed on all CRC datasets previously studied. As shown in **Table S13**, the positive correlation between LINC00483 and CTNNA1, HIGD2A, and MIER3 represents a common feature of most CRC expression datasets.

## Discussion

In this work we identified LINC00483 as a long non-coding RNA with a potential role of tumor suppressor in CRC. By screening several CRC datasets, we found that LINC00483 expression correlated with several protein-coding genes whose dysregulation was associated with CRC. Expression analysis performed on CRC biopsies compared to normal adjacent tissues, revealed a statistically significant downregulation of LINC00483, as well as its low expression levels in metastatic CRCs compared to non-metastatic primary tumors. This reduced expression associated with CRC was corroborated by an additional screening of all cancer datasets deposited in the R2 genomics repository showing that LINC00483 was steadily and specifically downregulated in CRC specimens from most of the CRC datasets available. Interestingly, LINC00483 expression seems to be particularly conspicuous in the physiologic intestinal epithelium and almost absent in other tissues, according to Fagerberg et al. (29), as reported in BioProject PRJEB4337. This would suggest that LINC00483 could play a specific role in the normal intestinal epithelium. Notably, aberrant expression of LINC00483 was reported for other cancers for which this lncRNA is considered an oncogene (30–32).

With the aim of understanding the functional involvement of LINC00483 in CRC biology, we evaluated its expression in CRC cells after the artificial block or induction of specific cancer related pathways. More specifically, LINC00483 levels increased after inhibition of MAPK signaling, which is usually overactivated in CRC cells and mainly responsible for cellular hyperproliferation (33). Notably, inhibition by U0126 treatment leads to G0/G1 arrest of cell cycle (34). Moreover, we observed an upregulation of LINC00483 also after serum starvation, an artificial experimental approach causing the exit of the cells from cell cycle (G0) and reducing the expression of genes responsible for cell cycle progression (e.g., HRAS, MYC), and components of the MAPK and PI3K pathways (35). These results would suggest that proliferative signals, including those mediated by ERK pathway, could transcriptionally or post-transcriptionally lower the amount of LINC00483. These considerations could explain the downregulation of LINC00483 in CRC biopsies (36, 37). When these signals were artificially inhibited, LINC00483 expression increased. Taken together, these data would suggest that LINC00483 could have a role in the G0/G1 cell cycle status of CRC cells, participating in a complex RNA-RNA network. Moreover, we don’t exclude that LINC00483 could play a role in stress conditions that subsequently would lead to G0/G1 block. This consideration could also explain the unaffected proliferation after the ectopic expression of LINC00483. LINC00483 exhibited low levels of expression also in metastatic CRC compared to primary tumors. This observation suggested a potential involvement of this lncRNA in one of the pathways underlying the metastatic process. It is now commonly accepted that EMT is an essential process for distant metastases formation. Although the EMT program was originally described as part of morphogenesis in embryonic development, it was later observed in several pathogenic events, such as fibrosis, wound healing, tumor progression, and metastases (38, 39). According to the importance that EMT activation takes on CRC metastatic development, we artificially induced EMT in CRC cells by using TGFβ-1 and IL-6, two well-known inducers of EMT in CRC, through different molecular cascades (28, 40). As expected, EMT induction by TGFβ-1 and IL-6 caused a decrease of LINC00483 expression, suggesting that it could play a negative role in EMT activation.

We found that enforced expression of LINC00483 in CRC cells induced a slowing down of the cell migration rate, suggesting that this lncRNA may exert a negative regulation of the motile phenotype, which is a hallmark of EMT impairment. On the other hand, LINC00483 did not induce any modulation of cell proliferation. Based on the latter observation, the upregulation of LINC00483 after inhibition of proliferative signals could be the result of a joint RNA-RNA network. Accordingly, ectopic expression of LINC00483 may be functionally effective for cell proliferation under certain molecular conditions, such as specific stoichiometric concentrations of its direct or indirect interactors (9).

In the last few years there has been increasing evidence of lncRNA function as ceRNA (competitive endogenous RNA) (41–43). Since LINC00483 is predominantly cytoplasmic, we hypothesized a “miRNA sponge” role for it. According to this model, miRNAs are sponged by lncRNAs: this prevents their binding to targeted mRNAs and their subsequent degradation. According to the miRNA sponge hypothesis for LINC00483, we computationally retrieved miRNAs that could simultaneously bind LINC00483 and mRNAs showing a positive correlation of expression with LINC00483 itself. By this approach, we reconstructed potential LINC00483-miRNA-mRNA molecular axes and evaluated the expression of the latter in different experimental conditions provoking the alteration of LINC00483. We focused our analysis on those mRNAs whose expression increased after the enforced expression of LINC00483, according to the miRNA sponge model. A number of mRNAs were modulated similarly to LINC00483 in the different treatments (i.e., TGFβ-1, IL-6, U0126, serum starvation), confirming their mutual positive correlation of expression with LINC00483 during the block of proliferation and EMT induction. CTNNA1 (catenin alpha 1), HIGD2A (HIG1 hypoxia inducible domain family member 2a), and MIER3 (MIER family member 3) showed an upregulation after inhibition of the MAPK pathway and a downregulation after EMT induction, in the same way as LINC00483. Analysis of both our CRC cohort and R2 genomics expression datasets showed the downregulation of CTNNA1, HIGD2A and MIER3 (also in association with the severe features of the tumor), but also an evident positive correlation of expression with LINC00483. CTNNA1 and MIER3 have a well-known tumor suppressor function. MIER3 was downregulated in colorectal cancer tissue compared to healthy colon mucosa (44). Peng *et al*. reported that MIER3 was significantly reduced in human primary CRC and was associated with CRC metastasis and poor prognosis (45). Upregulation of MIER3 expression significantly inhibited CRC cell proliferation, migration, and invasion *in vitro* and repressed tumor growth and metastasis *in vivo*. Moreover, MIER3 suppressed colorectal cancer progression and inhibited epithelial-mesenchymal transition (45). Several studies have shown the tumor suppressor role of CTNNA1 in different tumors (46–48). CTNNA1 expression was markedly lower in CRC tissues compared to adjacent normal mucosa and its overexpression significantly inhibited proliferation and migration of CRC cells (49, 50). Moreover, ablation of CTNNA1 induced alterations in cell–cell adhesion and enhanced cell migration (51). HIGD2A function is not well known. However, Ameri *et al*., showed that a member of the same family—HIGD1A (HIG1 hypoxia inducible domain family member 1A) interacts with the electron transport chain, modulating mitochondrial ROS production, oxygen consumption, and AMPK activity to promote survival during glucose starvation, while simultaneously decreasing tumor growth *in vivo* (52). HIGD1A may play an important role in tumor dormancy or recurrence mechanisms during tumor cell adaptation to extreme environments (52, 53). HIGD2A could play a similar role of tumor suppressor gene in CRC and be involved in tumor proliferation processes; however, its role in cancer biology remains to be elucidated. The other mRNA nodes of LINC00483 axes (ACVR1B, ARL4A, EIF6, EPCAM, PLS1, SRPK1, TPD52, VDR, ZDHHC9) could play a role in the regulation of the cell cycle, even if they were not modulated during artificial EMT induction. We suggest that the functional role of lncRNA molecular axes may not be ubiquitous but closely associated with specific molecular contexts (e.g., the interaction with other RNA molecules or proteins), which perturb the whole ceRNA network and, accordingly, the single lncRNA axis.

We identified HNF4α (hepatocyte nuclear factor 4 alpha) as a transcription factor that could potentially regulate LINC00483 expression. Indeed, by using a HNF4α inhibitor, we found that the expression levels of LINC00483 and all axis’s mRNAs strongly increased. Thus, we could speculate that HNF4α negatively regulates this lncRNA. HNFAα regulates the expression of many genes involved in several processes, such as development, metabolism, and epithelial-mesenchymal transition (54–56). Through mechanisms not completely understood, transcription from P1 or P2 promoters, combined with alternative splicing, potentially generates 12 different transcripts and 12 HNFAα protein isoforms (57). Each isoform performs a distinct function to regulate a specific subset of genes in a tissue-dependent manner (58, 59). There are contradictory reports on whether HNF4α acts as an oncogene or a tumor suppressor in different cancer models, including CRC (60–66). The HNF4A locus is amplified in CRC tumors and its overexpression is associated with specific subtypes of colorectal cancer (64, 67). HNF4α is known to act as a transcriptional activator (68, 69). However, post-translational modifications can influence the recruitment of coactivators and corepressors in order to modify the transcriptional influence of HNF4α on its target genes (70, 71). Schwartz *et al*. investigated the putative role played by HNF4α in CRC by evaluating the effect of HNF4α antagonists and HNF4α small interfering RNA (siRNA) on CRC growth and proliferation in cultured CRC cells and xenotransplanted nude mice *in vivo*. These molecules are shown to inhibit growth and proliferation of HT29 and Caco2 CRC cells (61). Based on our data, we propose that HNF4α could exert an oncogenic role by inhibiting LINC00483 expression in CRC. Accordingly, a gain of function of oncogenic HNF4a may be involved in downregulation of LINC00483. Interestingly, a potential target of HNF4α is the well-known tumor suppressor PTEN (72), which counteracts the PI3K/Akt signaling cascade and controls cell proliferation/invasiveness (73–75). However, LINC00483 downregulation in CRC could be due also to different aberrant transcriptional or post-transcriptional regulations.

Finally, our data suggest that LINC00483 plays a role of a tumor suppressor in CRC and through the miRNA sponge mechanism potentially controls the levels of a heterogenous set of mRNAs, which, in turn, may directly or indirectly modulate cell cycle and migration.

## Data Availability Statement

The original contributions presented in the study are included in the article/**Supplementary Material**. Further inquiries can be directed to the corresponding author.

## Author Contributions

MR and DuB designed and conceived the experiments with the critical collaboration of MP and CP. RC, GB, and LM obtained, characterized, and curated clinical data from patients’ biopsies. DuB and CB performed the experiments. FM, AC, RB, and DaB contributed to the acquisition, analysis, and interpretation of data. MR and DuB wrote the paper. MP and CP reviewed and edited the final version of the manuscript. All authors contributed to the article and approved the submitted version.

## Conflict of Interest

The authors declare that the research was conducted in the absence of any commercial or financial relationships that could be construed as a potential conflict of interest.

## Supplementary Material

Supplementary Figure 1 Schematic representation of lncRNA-miRNA-mRNA relationship based on the miRNA sponge model.

Supplementary Figure 2 Expression of LINC00483 and mRNAs associated with its molecular axes after 24 h serum starvation, with and without cell synchronization (complete data).
