# Supplementary figures and images for: LINC00483 Has a Potential Tumor-Suppressor Role in Colorectal Cancer Through Multiple Molecular Axes
Source: Front Oncol. 2021 Jan 20;10:614455. doi: 10.3389/fonc.2020.614455 (PMC7855711; doi:10.3389/fonc.2020.614455)

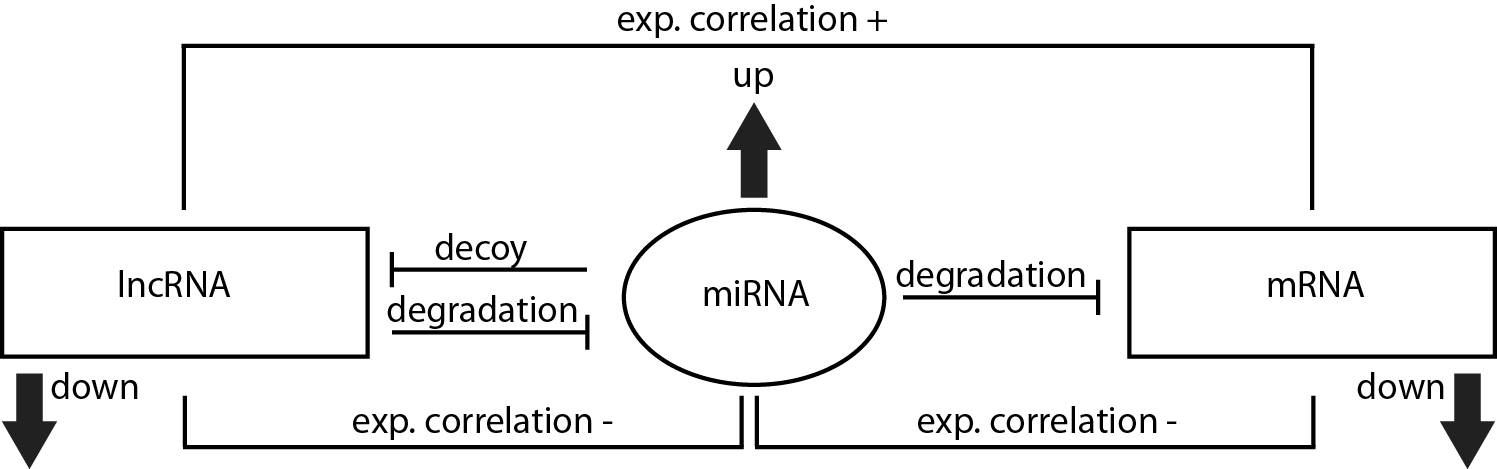

Supplement: Supplementary Figure 1 — Schematic representation of lncRNA-miRNA-mRNA relationship based on the miRNA sponge model. [file Image_1.jpeg]

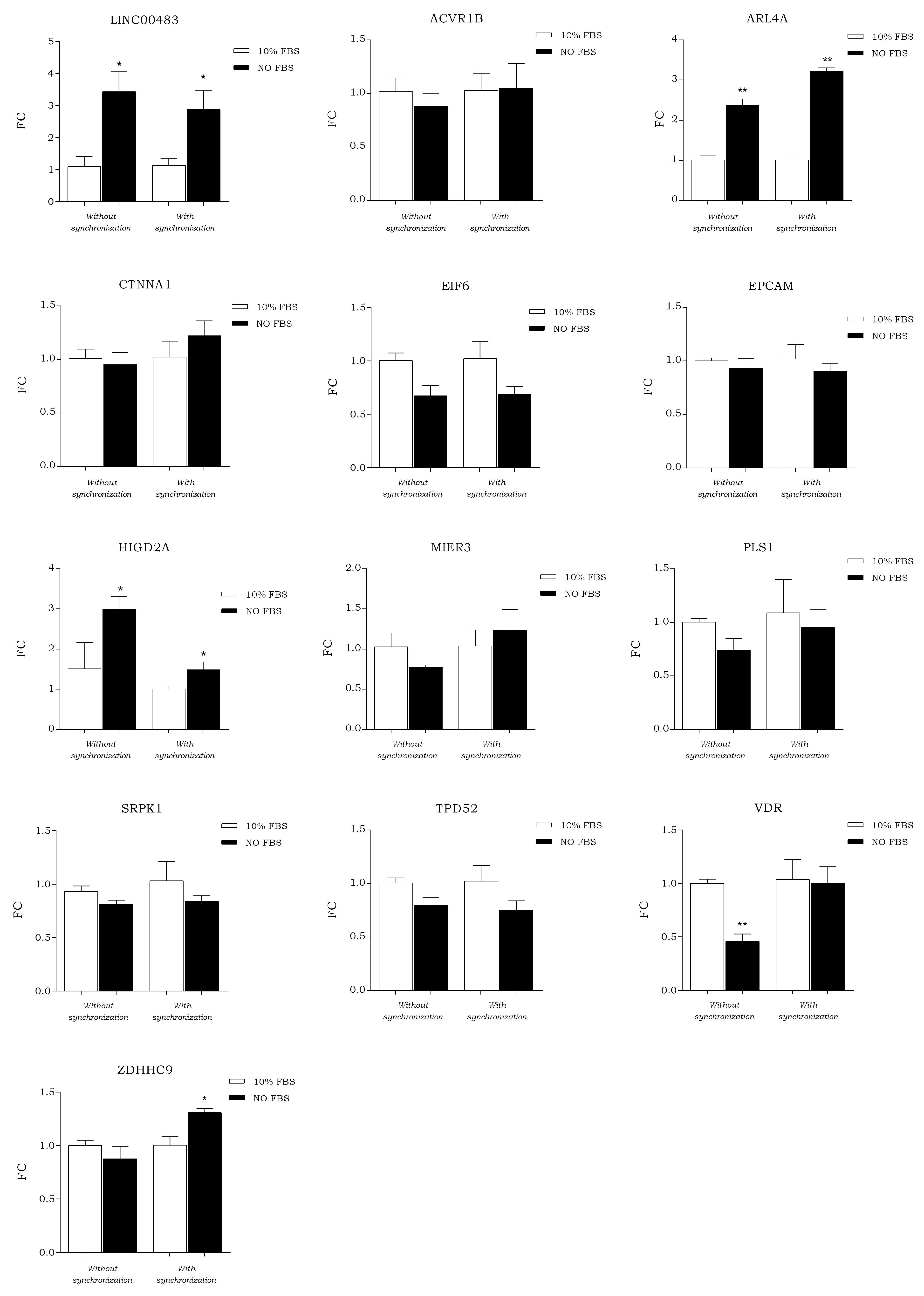

Supplement: Supplementary Figure 2 — Expression of LINC00483 and mRNAs associated with its molecular axes after 24 h serum starvation, with and without cell synchronization (complete data). [file Image_2.jpeg]
